# Comparisons with Amyloid-β Reveal an Aspartate Residue That Stabilizes Fibrils of the Aortic Amyloid Peptide Medin*

**DOI:** 10.1074/jbc.M114.602177

**Published:** 2015-01-22

**Authors:** Hannah A. Davies, Jillian Madine, David A. Middleton

**Affiliations:** From the ‡ Institute of Integrative Biology, University of Liverpool, Crown Street, Liverpool L69 7ZB, United Kingdom and; the § Department of Chemistry, Lancaster University, Lancaster LA1 4YB, United Kingdom

**Keywords:** Amyloid, Amyloid-beta (AB), Homology Modeling, Protein Misfolding, Protein Structure, Site-directed Mutagenesis, Dipolar Assisted Rotational Resonance, Rotational Echo Double Resonance, Salt Bridge, Solid-state NMR

## Abstract

Aortic medial amyloid (AMA) is the most common localized human amyloid, occurring in virtually all of the Caucasian population over the age of 50. The main protein component of AMA, medin, readily assembles into amyloid-like fibrils *in vitro*. Despite the prevalence of AMA, little is known about the self-assembly mechanism of medin or the molecular architecture of the fibrils. The amino acid sequence of medin is strikingly similar to the sequence of the Alzheimer disease (AD) amyloid-β (Aβ) polypeptides around the structural turn region of Aβ, where mutations associated with familial, early onset AD, have been identified. Asp^25^ and Lys^30^ of medin align with residues Asp^23^ and Lys^28^ of Aβ, which are known to form a stabilizing salt bridge in some fibril morphologies. Here we show that substituting Asp^25^ of medin with asparagine (D25N) impedes assembly into fibrils and stabilizes non-cytotoxic oligomers. Wild-type medin, by contrast, aggregates into β-sheet-rich amyloid-like fibrils within 50 h. A structural analysis of wild-type fibrils by solid-state NMR suggests a molecular repeat unit comprising at least two extended β-strands, separated by a turn stabilized by a Asp^25^-Lys^30^ salt bridge. We propose that Asp^25^ drives the assembly of medin by stabilizing the fibrillar conformation of the peptide and is thus reminiscent of the influence of Asp^23^ on the aggregation of Aβ. Pharmacological comparisons of wild-type medin and D25N will help to ascertain the pathological significance of this poorly understood protein.

## Teaser

**Background:** Aggregates of the 50-aa protein medin are the main constituent of aortic medial amyloid.

**Results:** Medin aggregation involves an aspartate residue analogous to Asp^23^ of the Alzheimer Aβ peptide.

**Conclusion:** There are striking similarities in the aggregation properties of medin and Aβ.

**Significance:** Mechanistic insights will assist future investigations of medin in the most common form of human amyloid.

## Introduction

**FIGURE 1.**
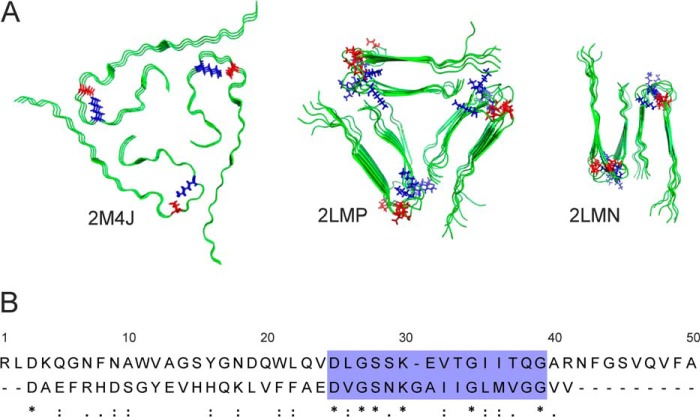
**Aβ(1–40) fibrillar structures and sequential similarities with medin.** *A*, structural models of Aβ(1–40) (viewed down the fibril axis) based on solid-state NMR restraints, taken from the Protein Data Bank files indicated. Asp^23^ and Lys^28^ are highlighted in *red* and *blue*, respectively. *B*, sequence alignment of medin (*top*) and Aβ(1–40) (*bottom*) performed using the LAlign server (29).

Approximately 30 proteins are known to form pathogenic amyloid or amyloid-like fibrillar networks in a wide range of human tissues (1) and are associated with diseases having high morbidity and mortality rates (2). Recent atomic and molecular level interrogations of fibrillar amyloid proteins have unveiled a variety of interactions, such as π-stacking of aromatic residues (3–6), van der Waals interactions (7), and salt bridges, that can stabilize the characteristic cross-β arrangement within the fibrils (8, 9). The 40- and 42-residue β-amyloid (Aβ) peptides associated with Alzheimer disease are structurally the best characterized of the amyloid proteins, and detailed molecular models have been assembled from NMR (9, 10), EPR (11, 12), and FRET (13, 14) constraints. The molecular repeat unit of the various known Aβ fibrillar morphologies consists of two β-strands separated by a turn through residues 25–29, which aligns the hydrophobic faces of the two cross-β segments (Fig. 1*A*).

Aortic medial amyloid (AMA),^2^ which is prevalent in over 97% of the Caucasian population over the age of 50, is primarily located within the medial layer of the aorta in close association with the elastic structures of the internal elastic laminae (15). It is thought that AMA may have a role in thoracic aneurysm and dissection (16). The polypeptide medin, a 50-residue cleavage product of the protein lactadherin, is the principal protein component of plaques (17). Although AMA is the most common form of localized amyloid, little is known about the biophysical and structural properties of fibrillar medin (15). Previous studies have demonstrated the 18–19 C-terminal residues of medin constitute an amyloid-promoting region, and a peptide, H_2_N-N^42^FGSVQFV-COOH (Med(42–49)), is capable of forming highly ordered fibrils within 48 h (3, 4). Structural studies of this fragment revealed β-sheets of hydrogen-bonded peptides in an in-register, parallel configuration with pairs of β-sheets stabilized by intermolecular π-π interactions between aromatic groups of amino acids Phe^43^ and Phe^48^ of opposing layers (6). Further solid-state NMR measurements indicated that this packing arrangement is not conserved in fibrils of full-length medin (5).

Medin has a 16% global sequence identity with Aβ and, strikingly, a high local sequence similarity within an amino acid stretch incorporating the turn region of Aβ from residues 25–29 (Fig. 1*B*, *box*). In this alignment, Asp^23^ and Lys^28^ of Aβ correspond precisely with Asp^25^ and Lys^30^ of medin. Asp^23^ and Lys^28^ of Aβ can form a stabilizing salt bridge that influences the geometry and mechanical properties of the fibrils (Fig. 1*A*) (9, 18–21). The region encompassing the Asp^23^-Lys^28^ salt bridge is also critical to the kinetic and structural elements of Aβ aggregation. Aβ(1–40) with a β-lactam bridge between Asp^23^ and Lys^28^ rapidly elongates into fibrils without a preceding lag phase and with a reduced critical concentration for fibril formation (22). Phosphorylation of Ser^26^ within the turn region has also been shown to prevent Aβ fibrillation (23). Importantly, mutations in the turn region of Aβ, including the Iowa mutation D23N, are associated with early onset of Alzheimer disease and cerebral amyloid angiopathy (24–28). The local sequence similarity of Aβ and medin may thus have implications for the fibrillar self-assembly mechanism of medin in AMA.

We investigate here whether Asp^25^ of medin influences the kinetics and pathway of protein self-assembly and the morphology of the aggregates, similarly to Asp^23^ of Aβ. The aggregation characteristics of the wild-type protein are compared with those of a model mutant, D25N, analogous to the naturally occurring, disease-linked D23N Aβ Iowa mutation. Solid-state NMR methods are used to investigate whether residues Asp^25^ and Lys^30^ of medin are capable of forming a salt bridge that influences fibril growth and morphology. A combination of biophysical measurements and computational modeling is used to provide the first working model of the medin fibrillar architecture.

## EXPERIMENTAL PROCEDURES

### 

#### 

##### Materials

Synthetic wild-type (WT) medin was purchased from Peptide Protein Research Ltd. (Fareham, UK) with uniformly ^13^C- and ^15^N-labeled amino acids Ala^13^, Asp^25^, and Lys^30^.

##### Sequence Alignment

Sequence alignment of medin and Aβ(1–40) was performed using the LAlign server (29). LAlign both provides a global sequence identity output and highlights areas of local similarity, which is ideal for identifying possible amyloidogenic motifs that may be missed with global alignments. The alignment was carried out using the BLOSUM50 matrix with a gap open penalty of −12 and a gap extension penalty of −2 with a threshold of 10.

##### Expression and Purification of Medin

Non-labeled and ^13^C and ^15^N isotope-labeled medin was expressed and purified as described by Davies *et al.* (30) and used for biophysical characterization and NMR studies. Medin Asp^25^ was mutated to Asn^25^ (D25N) using the site-directed ligase-independent mutation (SLIM) method as described by Chiu *et al.* (31) and confirmed by sequencing (GATC Biotech Ltd., London, UK). D25N was expressed and purified using the same procedures for producing WT non-labeled medin. Recombinant proteins were analyzed in 20 mm sodium phosphate, 150 mm NaCl, pH 7.4.

##### Biophysical Measurements

Thioflavin T (ThT) fluorescence assays were carried out on a Flexstation 3 microplate reader (Molecular Devices Ltd.). Experiments were carried out in triplicate in 96-well black-walled, clear bottomed microplates (Nunc). Data were recorded using bottom read mode, with excitation at 450 nm and emission at 485 nm. ThT solution was injected into the samples (20 μm medin) at the start of the read, to a final ThT concentration of 20 μm. The assay was conducted at 30 °C with no agitation. The ThT curves were fitted as described by Alvarez-Martinez *et al.* (32).

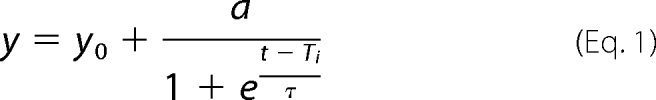
 where *y* represents the polymerization, *t* is time, *T_i_* is the inflection point of the sigmoidal, and the slope 1/τ is the rate of polymerization. The lag time can be calculated using the following equation,

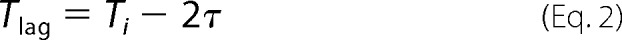
 Circular dichroism (CD) measurements were carried out at beamline B23 at Diamond Light Source (Oxford, UK). Freshly prepared medin was incubated at a concentration of 200 μm, at 30 °C, in the cuvette (121.000-QS, Hellma UK Ltd.) for the duration of the time course. Single scans were recorded every 2 h between 260 and 190 nm, using a slit width of 0.5 mm, a 0.5-cm path length, and a scan rate of 1 nm/s, over 44 h. The baseline was subtracted prior to secondary structure analysis. Analysis was carried out using Olis® GolbalWorks software. Data at each time point were subject to two fitting methods, CONTILL and CDSSTR, using either basis sets 8 or 11 (33–35). The best fit, as determined by the normalized spectral fit S.D. value, was selected, and the percentages of α-helix, β-sheet, turn, and random coil content were recorded.

Intrinsic fluorescence measurements were carried out on a Cary Eclipse Varian fluorescence spectrometer operating on a 20 μm medin solution at 30 °C. Tryptophan residues were excited at 279 nm, and the emission spectra were recorded between 300 and 400 nm with a band pass of 5 nm (36).

Transmission electron microscopy (TEM) was performed on medin after incubation for 50 h. Protein suspensions (10 μl) were loaded onto carbon-coated copper grids and negatively stained with 4% uranyl acetate. Samples were visualized on a Tecnai 10 electron microscope at 100 kV.

##### Cell Viability

Primary human aortic smooth muscle cells HAoSMC (Promocell, Germany) were plated on 96-well plates at 4,000 cells/well and grown for 48 h. Protein samples preincubated for 50 h at 20 μm were added to cells. Following incubation for 48 h, 10 μl of Cell Counting Kit-8 solution (Sigma-Aldrich, UK) was added and further incubated for 2 h, prior to measuring absorbance at 450 nm. The percentage of cell viability was calculated based on the absorbance measured relative to that of cells exposed to buffer alone.

##### Immunoblot Analysis

10 μl of peptide suspension was blotted onto nitrocellulose paper and allowed to dry. Blots were incubated with either A11 or OC primary antibodies (Merck Millipore) (1:1,000) for 1 h, washed, and then incubated with horseradish peroxidase-linked donkey anti-rabbit IgG secondary antibodies (GE Healthcare). Bound antibodies were detected using an electrochemiluminescence system (Merck Millipore) on carefully exposed film to avoid saturation.

##### Preparation of Medin Fibrils for Solid-state NMR Studies

Selectively labeled synthetic medin was lyophilized and subjected to three dissolution-evaporation cycles with hexafluoroisopropyl alcohol to break up any initial aggregates. The peptides were then dissolved in DMSO and added to double-distilled H_2_O to a final DMSO concentration of 10% (v/v) at a medin concentration of 200 μm and incubated with agitation at room temperature for up to 21 days. Uniformly ^13^C- and ^15^N-isotopically labeled recombinant medin was incubated at a concentration of 200 μm in buffer (20 mm sodium phosphate, 150 mm NaCl, pH 7.4) for 21 days with agitation at room temperature. The morphology of fibrils formed at 30 °C with no agitation and at room temperature with agitation was indistinguishable as assessed by TEM, but the latter method gave a much higher fibril yield and therefore was used to generate the NMR samples. The resultant fibrils were harvested by centrifugation at 21,000 × *g* for 1 h to generate a tightly packed pellet before being transferred to a zirconium 3.2- or 4-mm rotor with a Kel-F cap (Bruker, Coventry, UK).

##### Solid-state NMR Measurements

Dipolar assisted rotational resonance (DARR) NMR experiments were performed using a Bruker wide bore spectrometer operating at a static magnetic field of 20 teslas with a Bruker 3.2-mm triple resonance probe head in double resonance mode. Samples were maintained at −23 °C with a sample rotation frequency of 14 kHz ± 1 Hz. Experiments utilized a ^1^H 90° excitation pulse length of 2.5 μs, Hartmann-Hahn cross-polarization over a 1-ms contact time, 3-μs ^13^C 90° pulses, SPINAL proton decoupling at 100 kHz, and a 1.5-s recycle delay. The proton field was reduced to 14 kHz during DARR mixing times of 10 or 50 ms. Phase-sensitive spectra were obtained using time-proportional phase incrementation with 420 points in the indirect dimension. The spectrum at each *t*_1_ increment was the result of accumulating between 300 and 1024 transients. Frequency-selective rotational echo double resonance (FSR) solid-state NMR experiments were performed on hydrated fibrils using a Bruker Avance 400 spectrometer operating at a magnetic field of 9.3 teslas.

Frequency-selective rotational echo, double resonance (FSR) NMR experiments were performed on samples packed into a 4-mm zirconium rotor and rotated at the magic angle while maintaining the spinning rate automatically to within ± 1 Hz. All experiments utilized cross-polarization with an initial 4.0-μs ^1^H 90° excitation pulse, 1-ms Hartmann-Hahn contact time at a matched ^1^H field of 65 kHz, two-pulse phase-modulated proton decoupling (37) at a field of 85 kHz following cross-polarization, and a 1-s recycle delay. ^13^C-Observed FSR measurements with ^15^N dephasing were conducted using the pulse sequence described by Jaroniec *et al.* (38). The magic angle spinning frequency was 7,400 Hz. To observe ^13^C dephasing, a train of 82 or 122 non-selective 4-μs π pulses was applied at the ^15^N frequency every half rotor cycle, corresponding to total dephasing times of 5.5 and 8.2 ms, respectively. Frequency-selective 883-ms Gaussian pulses (defined by 1,000 points and truncated at 1% of the maximum amplitude) were applied in the center of the dephasing period. The frequencies of the Gaussian pulses were centered on the amide ^13^C and lysine ζ-^15^N resonance frequencies in order to selectively recouple the nuclear spins resonating at these frequencies. A second, control measurement was also performed at each dephasing time, omitting all ^15^N irradiation so as to observe the loss of ^13^C coherence resulting from processes not related to ^13^C-^15^N recoupling. Spectra were obtained by averaging eight blocks, each accumulated from 20,480 transients and alternating experiments with ^15^N irradiation and experiments without ^15^N irradiation so as to adjust for any drift in probe tuning during the long acquisition time. The extent of dephasing resulting from ^13^C-^15^N dipolar coupling (*S_D_*) was quantified as the peak intensity observed with ^15^N irradiation divided by the intensity observed in the absence of ^15^N irradiation. *S_D_* was translated into a ^13^C-^15^N distance using a C program written specifically for that purpose.

##### Simulation of DARR Spectra

DARR spectra were simulated using C programs written specifically for that purpose. Simulated DARR spectra were used to assess the secondary structure content of the experimental DARR spectra. Time domain signals were simulated as 512 × 512 matrices, and complex Fourier transformation was performed in two dimensions to obtain the frequency domain spectra. The ^13^C resonance frequencies used in the simulations were calculated using standard chemical shift values for α-helix and random coil (39) or using SHIFTX2 (40) back-predictions from structural models. ^13^C resonance frequencies were also taken directly from the simulated DARR spectra; only short range couplings between directly bonded spins were considered, and long range couplings were neglected to simplify the simulated spectra. Each free induction decay in the *t*_2_ dimension was modulated therefore by no more than three frequencies in *t*_1_ (*i.e.* depending on the number of bonded carbon atoms), which restricted the number of cross-peaks.

## RESULTS

### 

#### 

##### Fibrillization Invokes Large Scale Structural Changes in Medin

**FIGURE 2.**
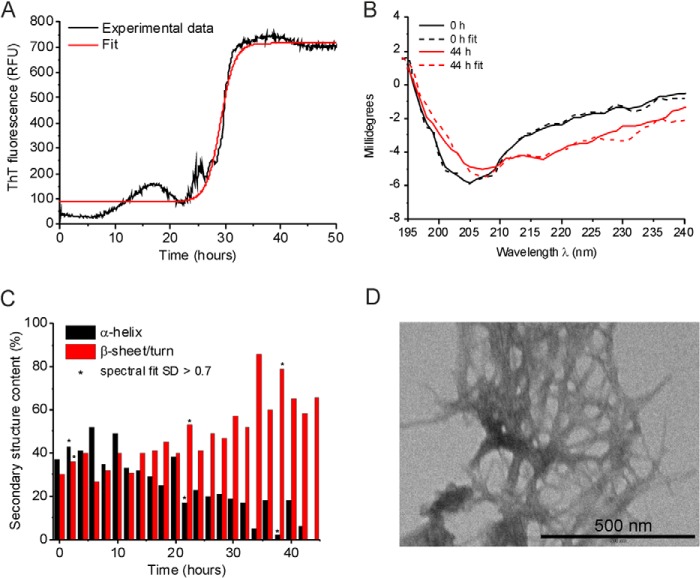
**Biophysical characterization of WT medin aggregation.** *A*, ThT fluorescence of medin aggregation. The mean fluorescence time course for three samples is shown. *B*, CD spectra of a medin solution obtained immediately after preparation (*black*) and after 44 h (*red*). *Dotted lines* represent the best fitting simulated spectra from which the secondary structure content was estimated. *C*, secondary structure changes during medin aggregation estimated from the CD spectra. *Asterisks* signify spectra for which the fitting process used to calculate secondary structure content resulted in S.D. of >0.07. *D*, TEM images of medin aggregates at 50 h. *RFU*, relative fluorescence units.

The aggregation properties of WT medin were assessed using several complementary techniques. ThT fluorescence indicated that medin (20 μm) aggregation occurs over 50 h (Fig. 2*A*). Analysis of the growth kinetics indicated that the elongation stage reached completion after ∼32 h with a growth rate of 1.28 h^−1^ (Table 1). The small reversible increase in fluorescence before the prominent enhancement is not typical of a lag period preceding nucleation and rapid elongation. Consequently, the fit to this part of the ThT data is poor, and it is not possible to measure with certainty the lag time or determine the time when irreversible fibril growth is initiated. The initial fluorescence increase may be due to a small population of transient, ThT-responsive intermediates, but the precise nature of these species is not known at this stage.

**TABLE 1:** **Comparison of WT and mutant kinetic parameters measured by ThT analysis** S.E. values for fits are given in parentheses. RFU, relative fluorescence units.

	WT	D25N
Maximum fluorescence intensity	716.07 (1.92)	567.31 (2.56)
Rate (h^−1^)	1.28 (0.04)	1.43 (0.08)
*R*^2^	0.985	0.951

Analysis of medin aggregation by CD (Fig. 2, *B* and *C*) shows a progressive change in the secondary structure over the 44-h time course. The onset of the structural transition occurs earlier than the large increase in ThT fluorescence, but this probably reflects the higher medin concentration required for the CD analysis (200 μm). Secondary structure content was estimated by applying four fitting regimes to the CD data: CONTILL with basis set 8, CONTILL with basis set 11, CDSSTR with basis set 8, or CDSSTR with basis set 11. All four regimes showed a conversion of α-helix to β-sheet over time but varied in the percentage contributions of the secondary structure. However, due to the dynamic changes occurring in the secondary structure and the solubility of medin over time, for each time point, we used the regime that gave the lowest S.D. and report the values recorded. Using this approach, the initial state is estimated to be predominantly helical and undergoes a transition toward ∼65% β-sheet (and ∼35% random coil) structure at the end point, consistent with amyloid formation (Fig. 2*C*). The fit at the end point was rather poorer than observed earlier, possibly because of precipitation of the insoluble fibrils. The structural conversion from α-helix to β-sheet broadly coincides with the aggregation time course observed in the ThT profile (Fig. 2, *A* and *C*). TEM of medin at the end of the time course (50 h) confirmed the presence of dense amyloid-like fibrillar networks (Fig. 2*D*).

##### Substitution of Aspartate 25 by Asparagine Stabilizes Medin Oligomers

**FIGURE 3.**
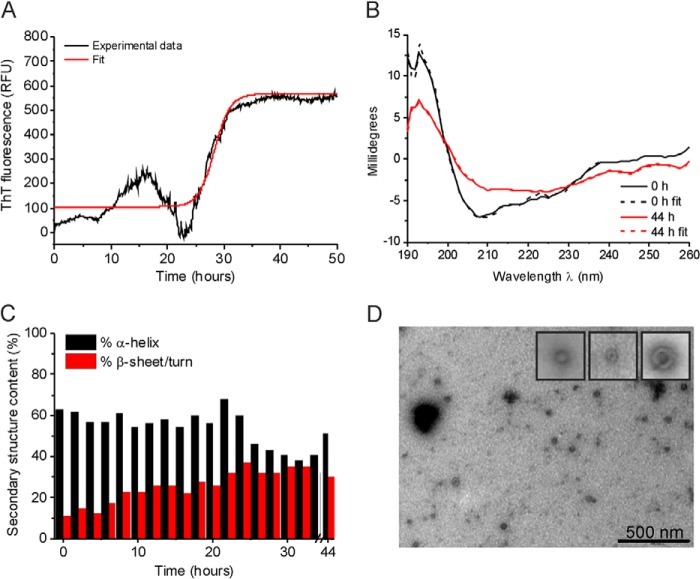
**Biophysical characterization of medin D25N aggregation.** *A*, ThT fluorescence of medin aggregation. *B*, CD spectra at the initial (*black*) and 44 h (*red*) time points with the best fitting simulated spectra (*dashed lines*). *C*, secondary structure changes during D25N aggregation estimated from the CD spectra. *D*, TEM images of medin aggregates at 50 h. *RFU*, relative fluorescence units.

**FIGURE 4.**
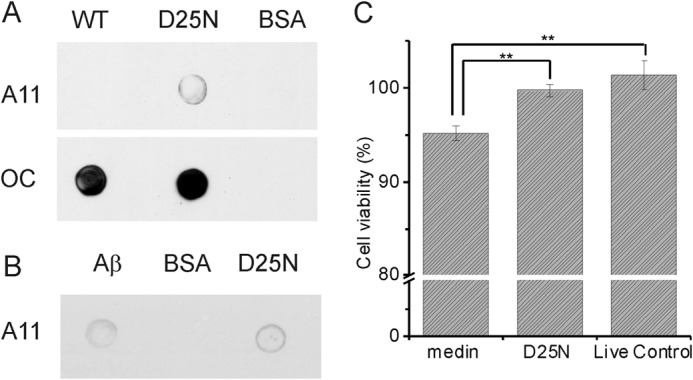
**D25N forms non-toxic prefibrillar oligomers.** *A*, immunoblot analysis of WT and D25N aggregates using A11 and OC conformer-specific antibodies. *B*, immunoblot analysis of D25N aggregates using A11 conformer-specific antibodies, including a stable Aβ oligomer (45) positive control and a BSA negative control. *C*, Cell Counting Kit-8 cell viability assay performed on human aortic smooth muscle cells. *, statistical significance (*p* < 0.01). *Error bars*, S.D.

In order to investigate the role of Asp^25^ in the self-assembly of medin, a model mutant with a single amino acid substitution, D25N, was generated. D25N showed a similar ThT profile to WT medin, albeit with a shorter lag time (26 h) and lower final ThT fluorescence (567 relative fluorescence units) (Fig. 4*A* and Table 1), reaching an apparent end point after ∼32 h. Like WT, D25N also exhibits an initial increase in fluorescence followed by a return to the baseline prior to the main fluorescence increase. As speculated above, this could represent a transient intermediate population that appears to be larger in relation to the final fluorescence than is observed for WT. The reversibility of the initial fluorescence hump may indicate that the intermediates undergo a structural rearrangement or disassemble into smaller species that are unresponsive to ThT. CD measurements indicate that although there is a general time-dependent shift toward a β-sheet structure, the mutant retains a much higher α-helical content than the WT protein after 50 h (∼50%) regardless of the regime and basis set used for the analysis (Fig. 3, *B* and *C*). Strikingly, TEM images of D25N after 50 h do not reveal any fibrillar structures, but small, spherical, concave structures are present that are similar in appearance to previously described oligomeric amyloid species (Fig. 3*D*) (41, 42). Hence, although the aggregation kinetics for WT medin and D25N appear to be similar as assessed by ThT, the mutant is impeded in the rate of assembly into fibrils.

The D25N species present after 50 h stain weakly with the A11 antibody that is reactive to prefibrillar oligomers regardless of protein sequence, whereas WT medin is A11-negative after 50 h (43) (Fig. 4*A*). Prefibrillar oligomers are defined as transient intermediates that are generally considered to undergo large scale concerted conformation changes to ultimately form fibrils (44). Hence, the mutation may stabilize prefibrillar oligomers of medin that, according to CD analysis, retain the initial helical conformation that WT medin loses during its unhindered assembly into fibrils. Both WT medin and D25N are stained much more strongly by the OC antibody that is reactive to both fibrillar oligomers and to fibrils (Fig. 4*A*) (43). In the case of WT protein, it is likely that the antibody reacts with the abundant protein fibrils, but for D25N, no fibrils were detected by TEM, so it is possible that OC is detecting fibrillar oligomers, which may also account for the relatively large ThT response. Stable Aβ oligomers were produced according to Haupt *et al.* (45) and used as a positive control for the A11 antibody. Bovine serum albumin (BSA) was used as a negative control (Fig. 4*A*).

The aggregated mutant and wild-type proteins were tested for their effects on human aortic smooth muscle cell viability. Following an equivalent incubation time (50 h), WT and D25N have significantly different cytotoxic effects (*p* < 0.01). The WT species show a small but significant (*p* < 0.01) increase in cytotoxicity relative to live control (Fig. 4*C*). The low level of toxicity for aggregated WT medin is consistent with medin fibril toxicity described previously (16, 46). By contrast, the D25N aggregates are not toxic to human aortic smooth muscle cells.

##### Assembly Pathways of Medin and D25N

**FIGURE 5.**
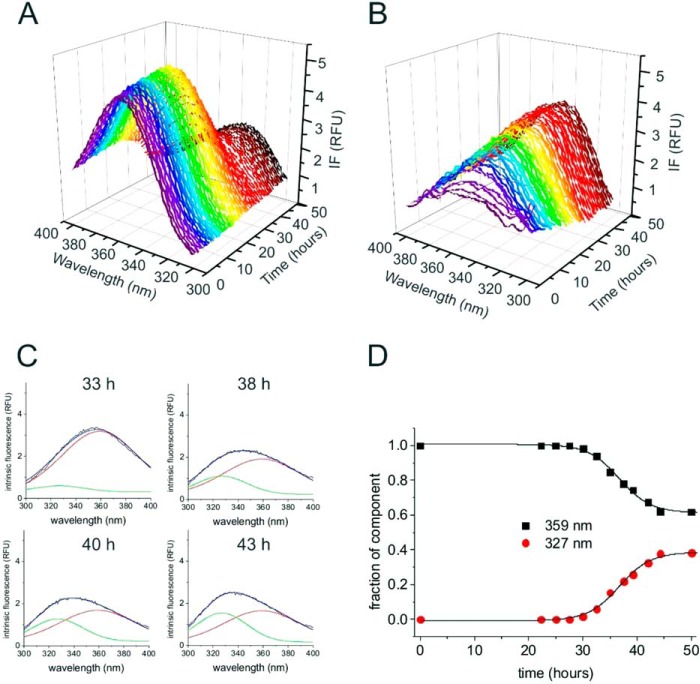
**Intrinsic fluorescence of medin tryptophan residues Trp^11^ and Trp^21^.** *A*, time course of fluorescence emission during WT medin aggregation. *B*, time course of fluorescence emission during D25N aggregation. *C*, deconvolution of the spectra for WT medin. Examples of spectra at the time points given (*black lines*) are superimposed with non-linear least squares fitted spectra (*blue lines*) approximated by two Gaussian components centered at 359 nm with a 60-nm width at half-height (*red line*) and at 327 nm with a 40-nm width (*green line*). The areas under the two components were the only variables. No further changes in the spectra were observed after 43 h. *D*, time course of the deconvoluted intrinsic fluorescence spectra of WT medin. The fractions of the two spectral components centered at 359 and 327 nm were calculated from the areas of the Gaussian curve fits. *RFU*, relative fluorescence units.

The D25N mutation may stabilize oligomeric species that are also formed by WT on-pathway to the fibrillar end-product, or the mutation may direct the assembly along a different pathway. Measurements of Trp^11^ and Trp^21^ intrinsic fluorescence provided some indication of the assembly pathways of the two medin peptides. WT medin (in the absence of ThT) gave an initial maximum fluorescence emission at around 360 nm, consistent with both tryptophan groups being exposed to the aqueous solvent (Fig. 5*A*). The emission is relatively constant for first 30 h, and then a gradual loss of overall fluorescence intensity occurs over ∼13 h, after which the fluorescence stabilizes again. The spectra were not consistent with a time-dependent shift of a single emission maximum wavelength but instead suggested that the initial species decreased over time as a new species emerged. Over a 50-h time period, the initial component at 355 nm decreased in intensity, and a second component emerged with an emission maximum around 330 nm, with both components being present at the end point (see below for further analysis). For D25N, no change in maximum fluorescence emission wavelength is observed over the 50-h time course, and, surprisingly, a substantial time-dependent increase in maximum intensity occurs at 342 nm (Fig. 5*B*). The fluorescence trend is consistent with both tryptophan side groups remaining exposed or becoming more exposed to water over the 50-h time course. The marked differences in the tryptophan fluorescence imply that the two medin peptides follow rather different assembly pathways.

One interpretation of the WT medin fluorescence profile is that a new species is formed over time with one of the tryptophan groups remaining solvent-exposed (the ∼359-nm component) and the other in a more hydrophobic environment (the ∼330-nm component) (47). A good fit to the initial (*t* = 0) spectrum could be obtained with a single Gaussian curve centered at 359 nm (not presented). Subtraction of the initial Gaussian curve (scaled appropriately) from the end point spectrum at 50 h yielded a difference spectrum that could also be fitted by a Gaussian function, centered at 327 nm (not presented). Good fits to all of the spectra at the intervening time points were then obtained by combining the two Gaussian curves in different proportions to give estimates of the contributions of the two components (examples given in Fig. 5*C*). The change in emission intensities for the two components mirrors the aggregation time course observed in the ThT fluorescence profile (Figs. 5*D* and 2*A*).

##### Evidence for a Asp^23^-Lys^28^ Salt Bridge in Wild-type Medin Fibrils

**FIGURE 6.**
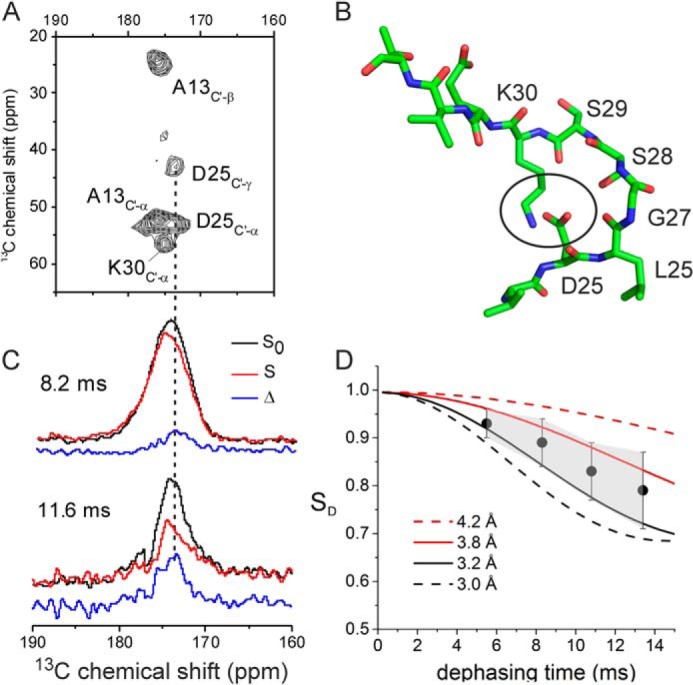
**Evidence for a salt bridge between Asp^25^ and Lys^30^ in medin fibrils.** *A*, amide region of a ^13^C DARR NMR spectrum of selectively labeled medin fibrils. *B*, a model of a medin monomer with an intermolecular salt bridge between the Asp^25^ and Lys^30^ side groups (*circled*) constraining the backbone in a turn. *C*, detection of ^13^C-^15^N dipolar coupling by FSR NMR analysis of the fibrils at 8.2 and 11.6 ms dephasing times. *Black*, control full-echo spectrum. *Red*, dephased echo spectrum obtained with a π pulse train at the frequency of Nζ for Lys^30^. *Blue*, difference spectrum (Δ). *D*, plot of observed FSR dephasing (*circles*) with simulated curves for different ^13^C-^15^N distances as stated. The *error bars* and *shaded region* represent the level of the noise. *Error bars*, S.D.

Asp^25^ clearly influences the aggregation pathway and morphology of medin, so we investigated whether a Asp^25^-Lys^30^ salt bridge, analogous to the Aβ Asp^23^-Lys^28^ salt bridge, influences medin self-assembly and is present in the insoluble protein fibrils. In order to test for the presence of a salt bridge, fibrils were prepared from synthetic medin containing uniformly ^13^C/^15^N-labeled Asp^25^ and Lys^30^ to enable measurements of distance-dependent ^15^N-^13^C dipolar couplings between the two residues by FSR solid-state NMR. The protein was also uniformly ^13^C-labeled at Ala^13^ to probe the local structural environment from the measured chemical shifts. A ^13^C DARR spectrum of the fibrils (at a 50-ms mixing time) was assigned unambiguously to all carbon sites of the labeled amino acids (amide region shown in Fig. 6*A*), and the ^13^C chemical shifts are summarized in Table 2. The values for the Cα, Cβ, and carbonyl (C′) shifts for the three residues are all consistent with these regions of the sequence adopting a β-strand conformation (39). A Asp^25^-Lys^30^ salt bridge would require a necessarily short (∼4.0 Å) separation of the carbon and nitrogen atoms of the amino acid COO^−^ and NH_3_^+^ groups (Fig. 6*B*). FSR measurements were carried out under conditions to detect a dipolar interaction selectively between the amino ^15^Nζ of Lys^30^ and Cγ of Asp^25^ if the distance between them were constrained by a salt bridge. The dipolar interaction was monitored by measuring the intensities of the peak envelope in the carbonyl region (from 167 to 183 ppm) of the ^13^C spectrum (Fig. 6*C*). The ratio of the peak intensities measured with radiofrequency pulses applied at the ^15^N frequency (*S*) and the measured intensities in the absence of pulses (*S*_0_) is, in general, proportional to the ^13^C-^15^N interatomic distance. Here, a progressive decrease in *S*/*S*_0_ is observed as the dephasing time increases (Fig. 6, *C* and *D*). The reduction in intensity is not uniform across the entire carbonyl region, and a difference spectrum (Δ) indicates that the loss of peak intensity is centered at 173.0 ppm, close to the resonance frequency of Cγ for Asp^25^ (Fig. 6*C*). The FSR data were compared with simulated *S*/*S*_0_ curves corresponding to different ^13^C-^15^N distances (Fig. 6*D*, *dashed* and *dotted lines*, respectively). A correction factor of 0.25 was applied to the simulated curves to adjust for the Asp^25^ (backbone), Lys^30^, and Ala^13^ signals that overlap with the Asp^25^ Cγ peak in the carbonyl region. When taking the signal/noise ratio into consideration (indicated by the *error bars* and *shaded region*), the data fall within the range bounded by curves representing ^13^C–^15^N distances of 3.8 and 3.2 Å. These values lie within the 4-Å limit for oppositely charged groups that commonly defines a salt bridge (48). Furthermore, these values are remarkably similar to the Asp^23^/Lys^28^ calculated distance reported for some Aβ fibril morphologies (10). Although there are uncertainties in the accuracy of these measurements arising from the overlapping signals in the carbonyl region, the FSR data together with the observations of D25N support the argument that a Asp^25^-Lys^30^ salt bridge stabilizes medin fibrils. It is not possible at this stage to determine whether the salt bridge is intramolecular, intermolecular, or a mixture of both.

**TABLE 2:** **Summary of ^13^C chemical shifts (in ppm) for medin fibrils uniformly labeled at Ala^13^, Asp^25^, and Lys^30^** Values were taken from a DARR spectrum with a 10-ms mixing time. Values in parenthesis are mean values for these amino acids in a β-sheet conformation, taken from Wang and Jardetzky (39).

Residue	Position					
	C′	Cα	Cβ	Cγ	Cδ	Cζ
	*ppm*					
Ala^13^	175.7 (175.7)	51.2 (51.4)	22.9 (21.6)			
Asp^25^	173.8 (174.1)	53.4 (53.2)	41.3 (42.9)	173.2		
Lys^30^	175.1 (174.8)	55.7 (55.5)	36.8 (34.7)	25.2	29.7	42.5

##### Structural Analysis of Wild-type Medin Fibrils

**FIGURE 7.**
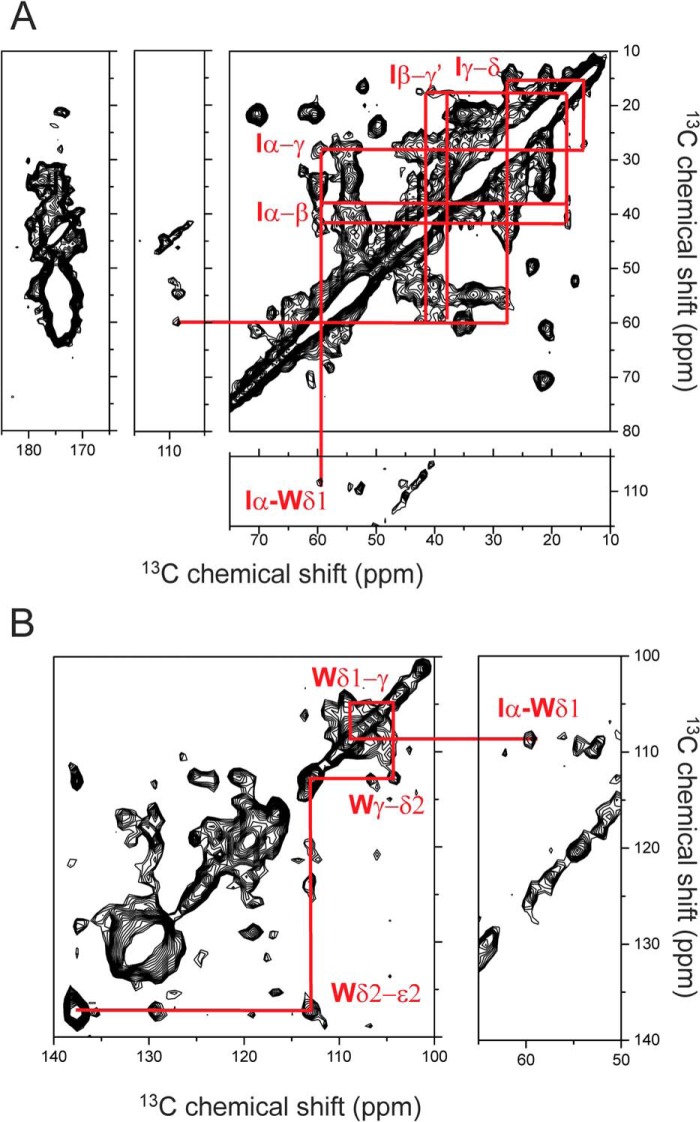
**Regions of a ^13^C DARR NMR spectrum of uniformly ^13^C-labeled WT medin fibrils.** *A*, amide, aromatic, and aliphatic regions highlighting connectivities within the isoleucine spin system and the putative coupling between isoleucine Ile^35^ and/or Ile^36^ and tryptophan Trp^11^ and/or Trp^21^. *B*, aromatic region highlighting tentative assignments for the tryptophan spin system.

A ^13^C DARR solid-state NMR spectrum of uniformly ^13^C/^15^N-labeled medin fibrils exhibits peaks that are rather broad and suggestive of local structural disorder or fibrillar heterogeneity (Fig. 7). Nevertheless, several intraresidue cross-peaks can be identified as well as an interresidue cross-peak indicative of long range coupling between tryptophan residue Trp^11^ and/or Trp^21^ and isoleucine residue Ile^35^ and/or Ile^36^ (Fig. 7). This cross-peak remained visible in a DARR spectrum of fibrils prepared from labeled medin diluted with a 3-fold excess of unlabeled medin, although it was not possible to determine the relative cross-peak intensity because of lower signal/noise ratio (data not shown). Dilution tends to abolish cross-peaks arising from intermolecular couplings, and the data suggest that the Trp-Ile cross-peak arises from intramolecular coupling between tryptophan and isoleucine residues. However, higher (>5-fold) dilution would be necessary to attribute the cross-peaks to intramolecular couplings unequivocally, but this was not possible because the further signal reduction would require longer measurement times than were available.

**FIGURE 8.**
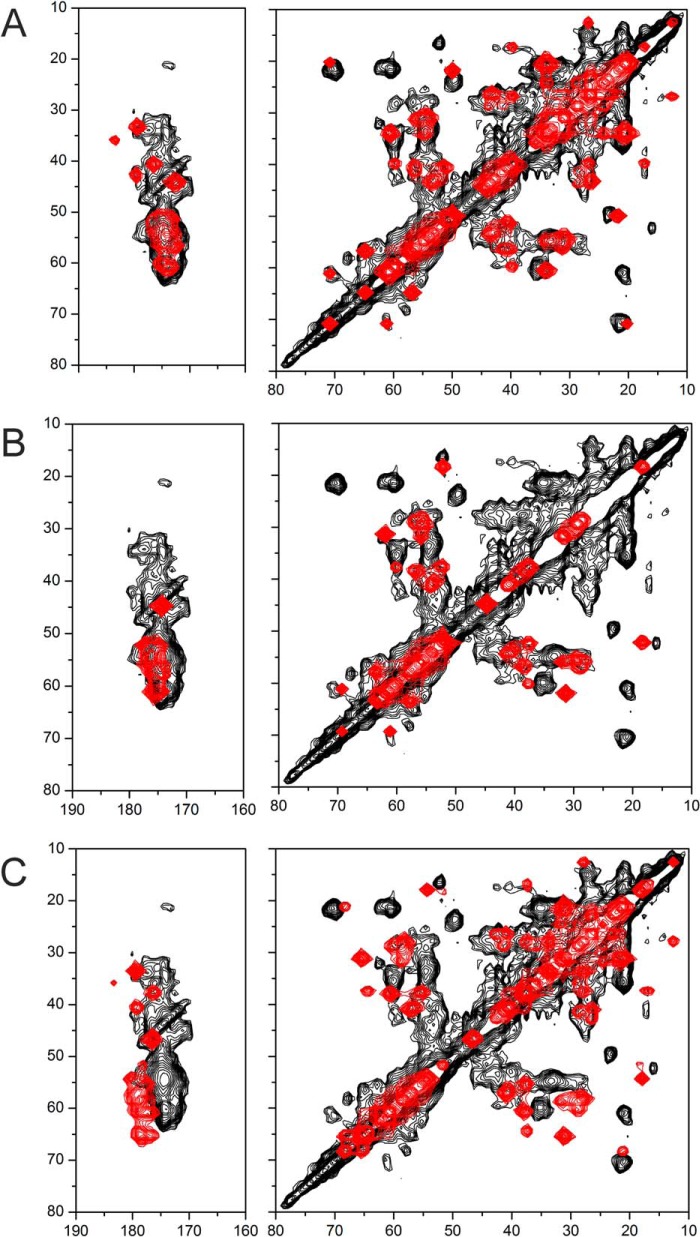
**Comparison of the DARR NMR spectrum of uniformly ^13^C,^15^N-labeled medin with simulated spectra (*red*) for 100% β-sheet (*A*), 100% random coil (*B*), and 100% α-helix (*C*).**

The spectrum was compared with simulated spectra generated from average Cα, Cβ, and C′ chemical shift values for medin residues in 100% α-helical or β-sheet conformations and as 100% random coil (Fig. 8). Similarities in the experimental and simulated spectra suggest that the fibrillar structure is a mixture of random coil and β-sheet elements (Fig. 8, *A* and *B*), but there is little evidence of any α-helical content (Fig. 8*C*). The shifts measured from a few characteristic Cα-Cβ cross-peaks are consistent with the two threonines (Thr^33^ and Thr^37^), some of the valines (Val^12^, Val^22^, Val^32^, Val^46^, and Val^48^), and some of the alanines (Ala^10^, Ala^13^, Ala^40^, and Ala^50^) occurring in β-sheet regions. However, at least one valine and one alanine (not Ala^13^) and at least one of the tryptophans (Trp^11^ and/or Trp^21^) occur in disordered regions.

**FIGURE 9.**
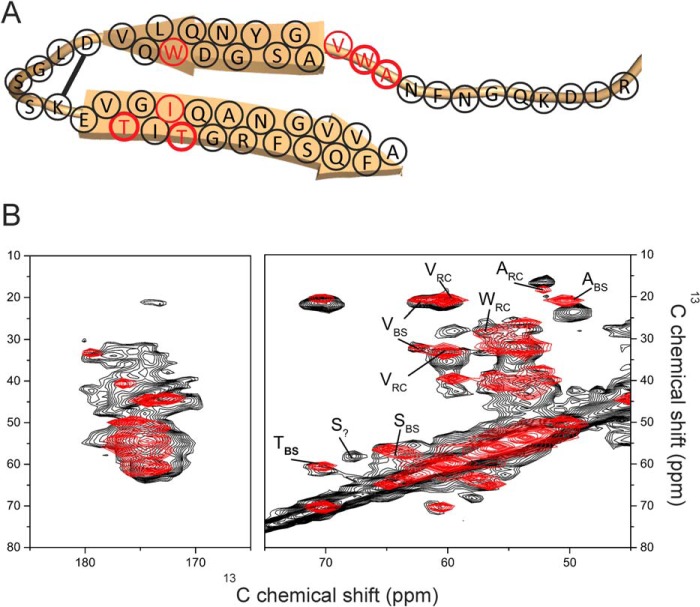
**Preliminary structural modeling of medin fibrils.** *A*, scheme of the proposed hairpin topology of the medin monomer constrained by a Asp^25^-Lys^30^ salt bridge. Residues Ala^10^, Trp^11^, Val^12^, Trp^21^, Thr^33^, Ile^34^, and Thr^37^ (highlighted in *red*) are discussed under “Results.” *B*, a DARR NMR spectrum of uniformly ^13^C-labeled fibrils (*black*) superimposed with a simulated spectrum (*red*) calculated from average chemical shifts for the amino acids in the model. All chemical shift values for the amino acids outside the β-sheet regions were taken from tabulated random coil values (39).

**FIGURE 10.**
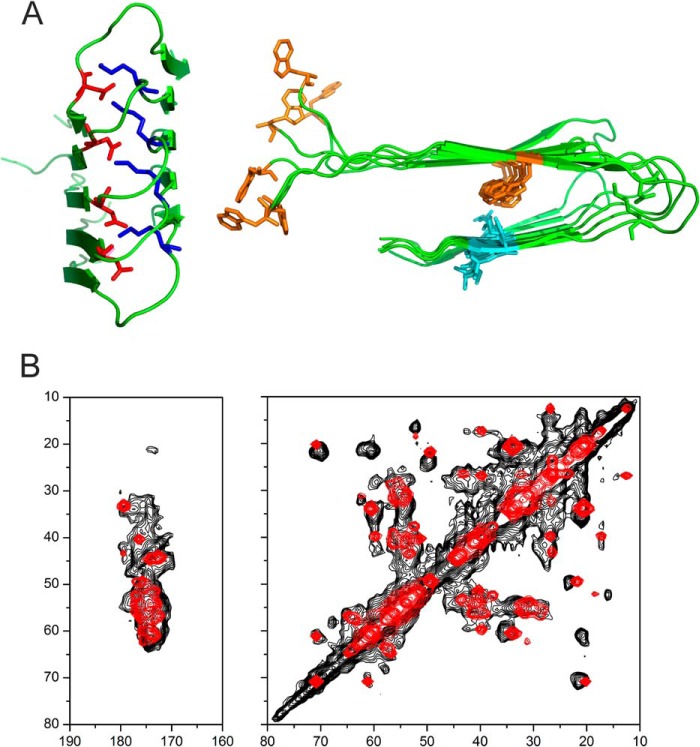
**Homology modeling of fibrillar medin.** *A*, model of medin fibrils created in Modeler using the Aβ(1–40) structural model (Protein Data Bank entry 2LMN) as a template and the sequence alignment in Fig. 1*B*. No further refinement of the model was performed. The *left view* (perpendicular to the fibril axis) shows the turn region stabilized by the salt bridge between Asp^25^ (*red*) and Lys^30^ (*blue*). The *right view* (down the fibril axis) shows Trp^11^ and Trp^21^ (*orange*) and Ile^35^ and Ile^36^ (*cyan*), with close packing of Trp^21^ and Ile^35^. *B*, simulated DARR spectrum (*red*) calculated from ^13^C chemical shifts predicted from the structural model using SHIFTX2, superimposed on the experimental spectrum (*black*).

Topological models of the fibrillar assemblies of Aβ, amylin, and others indicate a common hairpin-like structure (49). We propose, as a working model, a similar structure for the medin fibrils, with a disordered N-terminal region and two β-sheet segments separated by a turn stabilized by a Asp^25^-Lys^30^ salt bridge (Fig. 9*A*). The tentative divisions between the unstructured, β-sheet, and turn regions are based on the analysis of the DARR spectrum, with Ala^10^, Trp^11^, and Val^12^ in the unstructured region and Trp^21^, Thr^33^, Thr^37^, and the remaining alanines and valines in β-sheet regions (Fig. 9*B*). One serine Cα-Cβ cross-peak could not be accounted for by standard β-sheet, α-helix, or random coil chemical shift values (*labeled S*_?_ in Fig. 9*B*), possibly originating from Ser^28^ or Ser^29^ in the turn region. The 18 C-terminal residues alone are known to be highly amyloidogenic (4–6), and it is reasonable to model this region as one of the cross-β segments. Moreover, Trp^11^ is solvent-exposed, and Trp^21^ is buried within a hydrophobic cross-β interface close to Ile^35^, consistent with the fluorescence data and the DARR spectrum. A simulated DARR spectrum, generated from the predicted chemical shifts for this model, agrees well with the experimental spectrum (Fig. 9*B*, *red spectrum*). The proposed scheme in Fig. 9*A* is reminiscent of the Aβ hairpin structure (9, 10), and, interestingly, a homology model of medin, generated using the Aβ(1–40) 2-fold symmetry model (Protein Data Bank entry 2LMN) as a structural template and the sequence alignment in Fig. 1*B*, also allows for an Asp^25^-Lys^30^ salt bridge and close packing of Trp^21^ and Ile^35^ (Fig. 10*A*). A simulated DARR spectrum, calculated from the ^13^C chemical shifts predicted from this model using SHIFTX2, also agrees well with the experimental spectrum (Fig. 10*B*). This model will serve as a useful guide for the design of further experiments to test and refine the medin fibrillar structure.

## DISCUSSION

We report here the first systematic characterization of medin self-assembly. Under the conditions used here, WT medin undergoes a secondary structure rearrangement to form characteristic amyloid-like fibrils following a lag time of ∼27 h. This is consistent with previous reports of medin aggregation *in vitro* (4). Comparison of medin with the well characterized Aβ polypeptides led us to formulate a hypothesis of a stabilizing salt bridge in medin aggregates. Here, we have demonstrated, using computational modeling, CD, and solid-state NMR, that medin aggregates form predominantly β-sheet structures, consistent with a hairpin arrangement. Additional internuclear distance measurements suggest that a salt bridge at this location is feasible. Furthermore, removal of the aspartate residue at position 25 results in an altered self-assembly pathway suggesting that it has a key role in directing amyloid formation of medin.

The altered aggregation characteristics of D25N lead to a morphologically distinct species with a high α-helical content reminiscent of previously identified α-helical amyloid oligomers (50–52). Interestingly, the D25N species appear to be ThT-responsive (Fig. 3), yet TEM analysis shows only concave circular species, 35–100 nm in diameter (Fig. 4*D*), similar to other reported amyloid oligomers (53–55). No fibrillar aggregates were detected by TEM, and no visible precipitate was observed, although the presence of a small, highly ThT reactive population of fibrils cannot be ruled out entirely. The ThT-reactive oligomers may also occur as short lived intermediates on the assembly pathway of WT medin but, in the D25N mutant, are prevented from maturing into fibrils. Although we did not test for the presence of ThT-reactive, soluble oligomers of WT medin, the intrinsic fluorescence measurements (Fig. 5) suggest that the D25N oligomers are formed on a pathway different from WT assembly. D25N assembly gives rise to a substantial increase in tryptophan florescence, whereas the assembly pathway of WT medin gives a concomitant decrease in tryptophan fluorescence. The presence of oligomers was confirmed using A11 conformer-specific antibodies (Fig. 4*A*). It is often speculated that amyloid oligomers are the predominant cytotoxic species along the amyloid formation pathway. In light of our findings, we tested the cytotoxicity of D25N oligomer-like aggregates on human aortic cells. Interestingly, these aggregates were not significantly toxic to cells (Fig. 4*C*). D25N thus interferes with medin aggregation, appearing to prevent or impede the evolution of oligomers into fibrillar assemblies. It is possible that the aggregates formed by D25N represent an off-pathway intermediate (56). Recent work on Aβ has suggested that the presence of a salt bridge may be required for fibrillization, but may not be needed for oligomerization (23).

Asp^25^ appears to be influential in directing the self-assembly pathway of medin, reminiscent of the role that Asp^23^ plays in Aβ assembly. The Iowa Aβ mutant (D23N) completely alters the aggregation kinetics and fibrillar structure in familial, early onset Alzheimer disease (23). This mutation rapidly accelerates aggregation and results in enhanced toxicity and altered pathology (27). Conversely, other mutations within the turn region (G25P and E22V) have been shown to form non-toxic oligomers (57). Moreover, phosphorylation of Ser^26^ in the turn region of Aβ destabilizes the hairpin conformation (23), whereas constraint of Asp^23^ and Lys^28^ by a lactam bridge stabilizes the hairpin and enhances the aggregation rate (22). There is thus substantial evidence that formation and stabilization of the β-hairpin is important for self-assembly of Aβ. The results here together suggest that a salt bridge-stabilized β-hairpin is also a critical motif for medin to adopt a stable fibrillar conformation.

## CONCLUSION

Although D25N is not a known physiological mutant in AMA pathology, this work helps us to understand the mechanism by which medin aggregates. Results presented here highlight the similarities between medin and Aβ and emphasize that in addition to the cross-β structure, amyloid proteins can share additional structural motifs that can drive the aggregation pathway. These common motifs may provide information that can be targeted for future diagnostic and therapeutic development.

## Footnotes

2 The abbreviations used are:

AMA: aortic medial amyloid
ThT: thioflavin T
DARR: dipolar assisted rotational resonance
FSR: frequency-selective rotational echo double resonance
TEM: transmission electron microscopy.
